# Severe falciparum malaria in young children is associated with an increased risk of post-discharge readmission or death: A prospective cohort study

**DOI:** 10.21203/rs.3.rs-5104320/v1

**Published:** 2024-11-13

**Authors:** Robert O Opoka, Ruth Namazzi, Dibyadyuti Datta, Paul Bangirana, Andrea L. Conroy, Michael J. Goings, Kagan A. Mellencamp, Chandy C. John

**Affiliations:** Aga Khan University; Makerere University; Indiana University School of Medicine; Global Health Uganda (GHU) Research Collaboration; Indiana University School of Medicine; Indiana University School of Medicine; Indiana University School of Medicine; Indiana University School of Medicine

**Keywords:** severe malaria, post-discharge, readmissions, mortality, children

## Abstract

**Introduction:**

Few studies have described post-discharge morbidity of children with specific manifestations of severe malaria (SM) beyond severe malarial anemia or cerebral malaria.

**Methods:**

Children 6 months to 4 years of age admitted at Jinja and Mulago hospitals in Uganda, with one or more of the five most common manifestations of SM, cerebral malaria (n=53), respiratory distress syndrome (n=108), malaria with complicated seizures (n=160), severe malarial anemia (n=155) or prostration (n=75), were followed for 12 months after discharge, along with community children (CC) (n=120) recruited from the household or neighborhood of the children with SM. Incidence and risk of post-discharge readmission, death or outpatient clinic visits were compared between children with SM and CC.

**Results:**

312/551 (56.6%) of children with SM had one or more post-discharge readmission, compared to 37/120 (30.8%, p<0.001) of CC. Frequency of readmission was similar across all forms of SM. Compared to CC, children with SM had significantly higher risk of post-discharge readmission or death (adjusted hazard ratio (aHR) 2.06, 95% confidence interval (CI) 1.51–2.81, p<0.001), but a similar risk of outpatient malaria (aHR 1.30, 95% CI 0.97–1.74, p=0.08). 82% of readmissions in children with SM were due to malaria.

**Conclusions:**

In this malaria endemic region, children with the most common forms of SM had higher rates of post-discharge readmission or death than CC, and >80% of readmissions were due to malaria. Studies of post-discharge malaria chemoprevention are urgently needed for children with SM, to determine if this treatment can reduce post-discharge morbidity and mortality.

## Background

Severe *P. falciparum* malaria remains a leading cause of mortality in children, accounting for an estimated 272,000 deaths in children under 5 years worldwide in 2018 (1). Although severe malaria is a single disease entity, it presents in patients with a wide range of clinical manifestations. The common manifestations of severe malaria in Uganda, and in many other malaria endemic areas, are cerebral malaria (CM), respiratory distress syndrome (RDS), malaria with complicated seizures (MS), severe malarial anemia (SMA), and prostration. Together, these common manifestations of severe malaria affect about 6 million African children and account for > 75% of pediatric malaria admissions (2).

Despite the broad clinical typology of severe malaria, malaria research tends to focus on only two of its manifestations—CM and SMA. CM is the most lethal manifestation of severe malaria with an inpatient case fatality rate of 15–20% (3), whereas SMA is the most common presentation affecting 7.5–34% of the children that acquire severe malaria (4–6). Mortality and morbidity in SMA is not limited to the inpatient period alone; survivors suffer significant post-discharge morbidity which can lead to readmission or death. A Malawian study found a 12.6% post-discharge all-cause mortality rate among children with SMA after 18 months. Similar studies from Uganda and Kenya revealed that between 10–16% of SMA survivors died or were readmitted within 6 months after discharge from the hospital (7–9). Whether the other manifestations of severe malaria suffer similar rates of post-discharge morbidity and mortality is not well described.

In a prior study among children in Kampala, we compared post-discharge morbidity between children with SMA or CM and community children (CC) (10). Compared with CC, children with CM had a higher risk of outpatient sick visits and both children with CM and SMA had greater odds of post-discharge readmissions (10). However, children < 18 months of age were excluded from the study, although children < 18 months in this area may develop severe malaria and are more likely to get repeated malaria attacks (10). Further, only two manifestations of severe malaria were considered. The present study expands this research by documenting post-discharge morbidity or mortality across the five most common manifestations of severe malaria in young children in two areas of different malaria transmission.

In this study, we evaluated risk of readmission or death over a 12-month follow-up period in a cohort of young children 6 months to 4 years of age admitted at two tertiary hospitals in Uganda for one or more of the five common manifestations of severe malaria, compared to a cohort of community children of similar age, recruited from the same household or neighborhood as the children with severe malaria.

## Methods

### Study participants.

The study was performed at Jinja Regional Referral, Jinja and Mulago National Referral Hospital, Kampala, in eastern and central Uganda, respectively, as part of a larger study assessing neurodevelopmental impairment in children. The Ugandan Ministry of Health classifies Kampala as an area of low malaria transmission, while Jinja is classified as an area of moderate to high malaria transmission (11). Children meeting any of the pre-specified criteria for severe malaria (SM), as defined in Table S1, or community children (CC) were enrolled if they were between 6 months and 4 years of age and had no known chronic illness requiring medical care. For the SM group, children were recruited if either their blood smear or rapid diagnostic test (RDT) (SD Bioline Malaria Ag *P. falciparum*/Pan, Abbott, Chicago, Illinois, USA) result were positive for malaria from the performing clinical laboratory at each hospital. Microscopy was then repeated in our research laboratory and children with confirmed *P. falciparum* on blood smear in the research laboratory or with *P. falciparum* infection documented by RDT were included in analysis. The manifestations of severe malaria were graded in a hierarchical order based on severity of case fatality rates as follows: cerebral malaria (CM) > respiratory distress syndrome (RDS) > malaria with complicated seizures (MS) > severe malarial anemia (SMA) > prostration. Children meeting more than one criterion for severe malaria were included in the higher-order group. CC were recruited from the family or neighborhood of children with severe malaria. To recruit CC, parents of children with severe malaria were provided information about the study, asked if any eligible children were present in their extended family, and invited to bring the eligible children to the clinic for evaluation. Parents of children in the household compound of a child with severe malaria were also notified about the study during a home visit. CC were enrolled in the study if they met the criteria outlined in Table S1.

### Clinical, laboratory and demographic assessment.

At enrollment, all study children underwent a medical history and physical examination. Nutrition was assessed by height- and weight-for-age z-scores using the World Health Organization standards. A blood smear for malaria and quantification of density of parasitemia was done by two independent microscopists with a third reading if results were discrepant. For all children with severe malaria, malaria blood smears were repeated every 24 hours during the admission until the smear was negative. Human Immunodeficiency Virus (HIV) testing was done as per standard clinical care for all admitted children in Uganda. Socioeconomic status was measured using a previously described scoring system in healthy Ugandan children ≥ 5 years (12).

### Inpatient clinical care.

Children with severe malaria were managed according to the Ugandan Ministry of Health treatment guidelines current at the time of the study. These included intravenous artesunate treatment for at least 24 hours followed by a full dose of artemether-lumefantrine once the child was able to take it orally. All children with a hemoglobin ≤ 5 g/dL received a blood transfusion. Other medical problems were treated as per the standard protocols applicable at the study hospitals.

### Study follow-up.

All study children received an insecticide-treated bednet. Study children were asked to return hospital at one month (severe malaria group only) for evaluation of interim health, and at 12 months for neuropsychology testing. Caregivers were asked to bring their children back to study hospitals whenever the children fell sick during the follow-up period. Transportation costs were reimbursed for these visits, and all clinical care was provided free of charge. During each hospital visit, the children were assessed and managed by study clinicians as per national guidelines. All children who presented with a history of fever or had fever on examination had a malaria blood smear performed at the research laboratory as described above. At the end of the follow-up period, the vital status of study participants was noted and any deaths that occurred during the follow-up period were recorded. The primary outcome for this study was a composite of readmission or death.

### Statistical analysis.

Analysis was done using Stata v18 (StataCorp., College Station, Texas). Demographics among the two groups were compared using Pearson Chi-square or Fisher’s exact tests for categorical variables (where appropriate) or Wilcoxon rank-sum tests for continuous variables. The incidence and risk of study outcomes (readmissions, clinic visits) were compared between children with SM and CC using the Prentice-Williams-Peterson Total-Time (PWP-TT) approach for recurrent events. The PWP-TT approach is advantageous when assessing the risk of recurrent events as it allows the underlying risk of failure (and the effects of covariates) to vary between repeated events while simultaneously correcting for within-subject correlations of failure times over follow-up. In the context of severe malaria, the PWP-TT approach is also better suited than other risk models to analyze the risk of readmission or death given that it can model death as a failure event following repeated hospitalizations (13).

Results are reported as adjusted hazard ratios (aHR) with 95% confidence intervals (CI). Analyses were adjusted for age (years), study site, presence of HIV infection, and socioeconomic status (SES) at enrollment. All study participants contributed person-time to the analyses and were censored when they died, withdrew from the study, were lost to follow-up or at their 12-month follow-up visit. There was no significant interaction between study site and SM group for all outcomes (p > 0.05 for all) (Table S3), so all analyses used pooled data from both sites. Missing covariate data were modest (0.5% for HIV infection, 10.1% for SES). Multiple imputation using chained equations was used to impute missing data for these two covariates in Stata using the *mi impute* command. No outcome data were imputed. A comparison of the primary analysis according to treatment of missing data is provided in Table S4.

## Results

### Baseline characteristics

**Figure 1:**
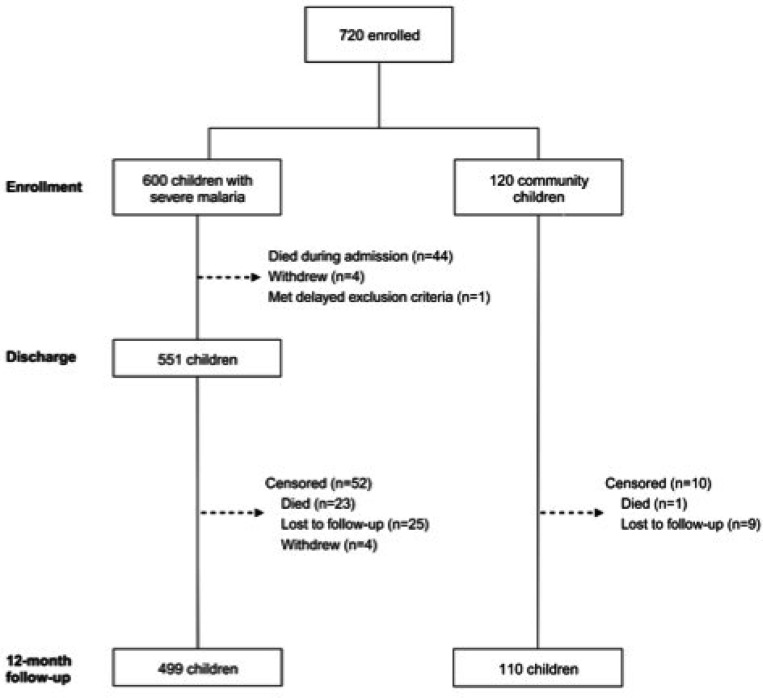
Flowchart of study participants from enrollment to 12-month follow-up

720 children were enrolled at the two sites, 600 children with severe malaria (SM) and 120 community children (CC). 49 children with severe malaria were excluded from the analysis because they withdrew from the study prior to discharge (n = 4), died during admission (n = 44) or met delayed exclusion criteria (n = 1). A total of 671 children (551 SM, 120 CC) were eligible for this study and included in the follow-up analysis (Fig. 1). 374 (55.7%) of the study participants were from the Kampala site, and the distribution of the different manifestations of SM were similar in the two sites (Table S2). Age and sex were similar between children with SM and CC (Table 1). All children with SM were positive by rapid diagnostic test (RDT), blood smear or both tests. 401 (72.9%) children with SM and 11 (9.3%) CC were blood smear positive for *P. falciparum* at enrollment, while all children with SM tested by RDT were positive for *P. falciparum*. All children with SM cleared parasitemia with artesunate and artemether-lumefantrine treatment prior to discharge. CC with parasitemia were treated with a full course of oral artemether/lumefantrine.

### Risk of readmission or death during 12-month follow-up

**Figure 2:**
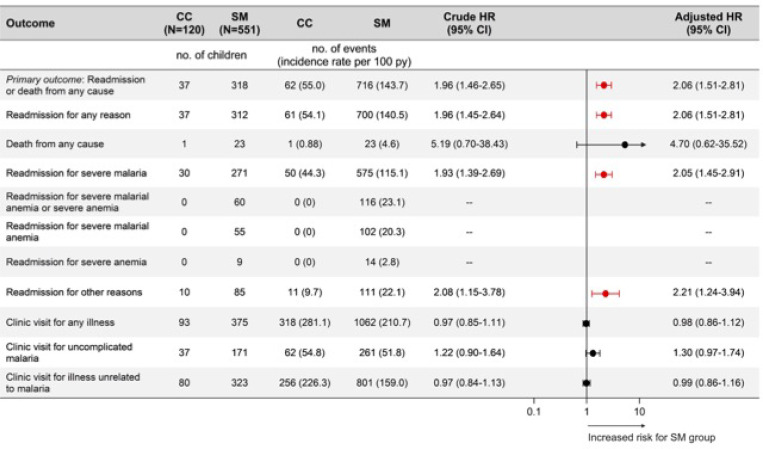
Incidence and risk of study outcomes between children with severe malaria and community children. Forest plot depicting adjusted hazard ratios (HR) and 95% confidence intervals (CI) from adjusted PWP-TT models for each recurrent outcome. Covariates in the models included age, study site, presence of HIV infection, and socioeconomic status at enrollment. aHR significant at p<0.05 depicted in red. aHR and 95% CI plotted on logarithmic scale. Abbreviations: SM, severe malaria; CC, community children; HR, hazard ratio; CI, confidence interval.

Overall, 778 events of readmission or death occurred in 355 of 671 children: 716 events in children with SM and 62 events in CC. Readmission or death occurred in 318 of 551 children with SM (57.7%) and in 37 of 120 CC (30.8%). A total of 166 children with SM and 13 CC had multiple events. Over the 12-month follow-up, risk of readmission or death was twice as high in children with SM than CC (aHR 2.06, 95% CI 1.51–2.81, p<0.001, Figure 2) adjusting for age, study site, presence of HIV infection, and socioeconomic status at enrollment. Similar results were seen in a model including only blood smear positive children with SM (aHR 1.92, 95% CI 1.38–2.66, p<0.001, Figure S1). Risk of readmission or death also differed significantly for each manifestation of SM compared to CC (Table 2). However, risk of readmission of death did not differ significantly between the individual manifestations of SM.

### Risk of readmission

Risk of readmission for any reason was significantly higher in children with SM compared to CC, mirroring the analysis of composite readmission or death (Figure 2). Risk of readmission for severe malaria and readmission for reasons unrelated to severe malaria or severe anemia were also higher in children SM compared to CC (aHR 2.05, 95% CI 1.45–2.91, p<0.001, and aHR 2.21, 95% CI 1.24–3.94, p=0.007, respectively). Similar results across these outcomes were seen when the analysis was restricted to blood smear positive children with SM compared to CC (Figure S1). Risk of readmission for any reason and readmissions for severe malaria were higher for children with each individual manifestation of SM than for CC (Table 2). However, only children with MS had a higher risk of readmission for reasons unrelated to severe malaria or severe anemia compared to CC.

### Risk of death

A total of 24 study children (3.6%) died during 12-month follow-up, 23/551 with SM (4.2%) and 1/120 CC (0.8%). The risk of death was higher in children with SM than in CC (aHR 4.70, 95% CI 0.62–35.52), though the difference did not reach statistical significance (p=0.13) likely due to only one death among CC (Figure 2). Children with RDS had the highest frequency of post-discharge deaths at 10 (9.3%), followed by children with SMA (n=8, 5.2%) and prostration (n=3, 4.0%) (Table 2). Most deaths (14/24, 58.3%) occurred in the community, and exact cause of death was difficult to ascertain, but often occurred after a short febrile illness. Seven (7) of the 10 follow-up deaths that occurred in-hospital were due to malaria-related severe anemia.

### Risk of outpatient clinic visits

468 (69.7%) study participants had outpatient clinic visits during the follow-up period, visiting the clinic 1,380 times, with 323 (23.4%) of these visits related to uncomplicated malaria. Although the incidence rates of outpatient clinic visits were higher in CC than in children with SM irrespective of the reason for the visit, the groups did not differ significantly in their risk of clinic visits over the 12-month follow-up (Figure 2). Comparable results were seen when the analysis was restricted to blood smear positive children with SM compared to CC (Figure S1). Risk of clinic visits was similar between the individual forms of SM compared to CC, but children with MS had a greater risk of clinic visits overall and clinic visits related to uncomplicated malaria compared to children with SMA (Table 2).

## Discussion

In this large prospective cohort study, we show that in areas of moderate and high malaria transmission, children with the five most common forms of severe malaria are all at significantly increased risk of readmission or death, and that readmission is overwhelmingly (>80%) due to malaria. In addition, we show that the risk of uncomplicated malaria is not increased in children with severe malaria, suggesting that children with severe malaria have underlying factors that predispose them to development of recurrent severe malaria. Overall, more than half (56.6%) of all children with severe malaria were readmitted post-discharge, which was similar to the readmission rate after severe anemia (45.9%) previously reported in the same setting (14). Our recent study of post-discharge malaria chemoprevention for children with severe anemia in malaria endemic areas showed a 35% reduction in readmission or death in this population (15). The present study suggests that trials of post-discharge malaria chemoprevention should be conducted in children with severe malaria, as the study suggests that malaria chemoprevention might markedly reduce post-discharge readmission in children with all forms of severe malaria, as it did in children with severe anemia.

Our study shows that the effects of clinical malaria extend beyond the initial inpatient period. Prior research first uncovered the post-discharge impact of CM. Studies found that 11 to 26% of children with CM were discharged with gross neurological deficits (16–18), while a further 25% developed long-term cognitive impairments, or behavioral or mental problems (3, 19–23). Later, SMA was shown to be associated with nearly two post-discharge deaths for every inpatient mortality (7, 8, 10), and was also shown to be associated with long-term cognitive and behavioral impairment (23, 24). Our study expands on this research by showing that other common manifestations of severe malaria are associated with marked post-discharge morbidity as well.

Malaria readmissions were increased (aHR 2.05), and risk of death was also increased (aHR 4.70), though the latter difference did not reach statistical significance. Among children with a known cause of death, most were due to severe malarial anemia (7 of 10, 70%). Together, the risk of readmission or death was greatly increased in children with each form of severe malaria compared to the community children. Our study data suggest that, although different manifestations of severe malaria impact children’s health distinctively, the effects of the disease may last for months after hospital discharge irrespective of the manifestation. Today, most clinical care is limited to the inpatient period. Our findings suggest that care should extend well beyond discharge.

In this study, repeat malaria infections were the most common reason for readmissions and/or death in the post-discharge period. All patients with severe malaria were treated according to clinical guidelines using intravenous artesunate followed by artemether/lumefantrine and had documented clearance of parasitemia (negative blood smear) before discharge. Anti-malarial studies in Uganda show that artesunate is still effective for rapid parasite clearance (25). Artemether/lumefantrine has also been shown to be effective at clearing residual parasites during the immediate post-discharge period (26). In addition, during the follow-up period, insecticide treated bed nets were used by the majority of children with severe malaria and CC similar (over 80% in both groups). We are currently conducting analysis to determine whether the follow-up visits for malaria were due to reinfection or recrudescence (26, 27). Further studies are needed to examine what factors predispose children with severe malaria to post-discharge morbidity in order to design effective interventions after children leave the hospital.

Whatever the pathological mechanisms, the effects of the initial episode of severe malaria appear to predispose the child to repeated episodes of severe malaria in the immediate post-discharge period. In this study, the curve of post-discharge events was steep in the first 3 months and began to plateau by month 6. More than two-thirds of the outpatient visits and more than 80% of the readmissions and deaths happened in the first 6 months, suggesting this critical time is when children with severe malaria are most vulnerable to recurrent illness, including recurrent severe malaria. During this period of incomplete recovery, children remain susceptible to repeat malarial attacks, including repeated episodes of severe malaria. While children with severe malaria are also vulnerable to uncomplicated malaria in this follow-up period, they are at no more risk than community children without severe malaria for uncomplicated malaria. Instead, children with severe malaria appear to have specific increased vulnerability to repeated severe malaria. Furthers studies are needed to uncover the precise mechanisms that make children with severe malaria vulnerable to repeated episodes of severe malaria in the first 6 months after discharge. What is clear, however, is that children with severe malaria remain vulnerable beyond the inpatient period, and that interventions to decrease post-discharge readmission in children with severe malaria are urgently needed.

Phiri and colleagues showed that provision of malarial chemoprevention with artemether lumefantrine in the first 3 months was associated with 31% reduction in morbidity and mortality in children with SMA (28). Recently, we completed a larger study in Kenya and Uganda that showed that in children with severe anemia, 85% of whom had malaria, a monthly treatment course of dihydroartemether-piperaquine given for 3 months was associated with 35% reduction of all-cause readmission or death over 6 months (15). In the first 3 months, the reduction was even greater at 70%. Malarial chemoprevention was most effective in reducing all-cause readmissions or mortality in the first 3 months in children who had malaria-related anemia (15). The study data led to a change in World Health Organization guidelines, which now state that a full therapeutic course of anti-malarial medicine should be given at discharge to children admitted to hospital with severe anemia living in settings with moderate to high malaria transmission (29). The present study adds to these findings, showing the severe malaria, even in the absence of anemia, is a strong risk factor for readmission or death.

The overall rate of readmissions observed in this study population is of public health concern. Although more children with severe malaria were readmitted than CC, it is concerning that nearly a third (30.8%) of all CC had at least one admission during the follow-up period. Over half (58.3%) of the deaths occurred in the community before the caretakers could bring the children back to hospital. This suggests that health issues are compounded by challenges in health services that need to be addressed. Future research should attempt to establish the reasons for the high post discharge morbidity in this region to inform implementation of comprehensive measures to improve children’s health outcomes.

Strengths of this study include the prospective study design encompassing two sites with different malarial transmission intensities and population characteristics (e.g., urban vs rural) and a control group. In addition, children with severe malaria were clinically diagnosed at admission and post-discharge morbidity was carefully documented over time. We therefore believe that our findings are reflective of the post-discharge morbidity in malaria endemic regions, and the high burden of malaria infections in the follow up period point to the urgent need for malarial chemotherapy beyond the admission period.

## Conclusion

In conclusion, we found that the rate of readmission or death was high in all of the 5 most common types of severe malaria during the 12 months after discharge. Children with SM had a two-fold increased risk of readmission or death that was largely due to repeat malarial infections in the post-discharge period. Further evaluation of post-discharge malarial chemoprevention for all children with severe malaria is needed in settings where it is not presently being implemented to determine the level of its efficacy for prevention of readmission or death after severe malaria.

## Supplementary Material

Supplement 1

## Figures and Tables

**Table 1:** Demographic and clinical characteristics of study participants at enrollment

	SM (N = 551)	CC (N = 120)	P value^a^
**Demographic characteristics**			
Age (years)	2.0 (1.4, 2.9)	2.2 (1.4, 3.1)	0.53
Sex (male)	317 (57.5)	64 (53.3)	0.40
Site (Kampala)	307 (55.7)	67 (55.8)	0.98
Duration of hospitalization (days)	4.0 (3.0, 5.0)	--	--
Used insecticide treated bednet last night^b^	416 (84.6) (492)	93 (85.3) (109)	0.84
SES score	10 (8, 14) (494)	11 (9, 15) (109)	0.06
**Clinical characteristics**			
Stunted (HAZ ≤ −2)	129 (23.4)	36 (30.0)	0.13
BS positive for malaria	401 (72.9) (550)	11 (9.3) (118)	< 0.001
Parasite density among BS+ (parasites/uL)	59018 (5702, 211881)	3181 (1198, 12915)	< 0.001
RDT positive for malaria	538 (100.0) (538)	29 (25.2) (115)	< 0.001
HIV positive	13 (2.4) (549)	0 (0) (119)	0.14

Abbreviations: SM, severe malaria; CC, community children; SES, socioeconomic status; HAZ, height-for-age z-score; BS+, blood smear positive; RDT, rapid diagnostic test.

Data presented as n (%) or median (IQR). If data were missing, the non-missing N is listed in final parentheses.

a P values obtained from Wilcoxon rank-sum tests for continuous variables, and Pearson’s Chi-square or Fisher’s exact tests for categorical variables (where appropriate).

b Measured at 6-month follow-up visit.

**Table 2.** Incidence and risk of recurrent readmissions and clinic visits between severe malaria manifestations and community children

Outcome	CC (N=120)	CM (N=53)	RDS (N=108)	MS (N=160)	SMA (N=155)	Pros (N=75)
no. children with event, no. of events (incidence rate per 100 py) aHR compared to CC (95% CI)^a^						

*Primary outcome*: Readmission or death from any cause	37, 62 (55.0)	31, 55 (114.4)	69, 184 (193.7)	90, 169 (114.9)	86, 217 (155.7)	42, 91 (132.5)
		**1.92 (1.33–2.78)**	**2.40 (1.70–3.39)**	**1.87 (1.33–2.63)**	**2.21 (1.58–3.08)**	**1.84 (1.26–2.70)**

Readmission for any reason	37, 61 (54.1)	31, 55 (114.4)	65, 175 (184.2)	90, 168 (114.2)	85, 213 (152.8)	41, 89 (129.6)
		**1.96 (1.35–2.84)**	**2.32 (1.64–3.28)**	**1.90 (1.35–2.68)**	**2.23 (1.60–3.11)**	**1.84 (1.26–2.70)**

Death from any cause	1, 1 (0.88)	1, 1 (2.1)	10, 10 (10.3)	1, 1 (0.67)	8, 8 (5.7)	3, 3 (4.3)
		2.51 (0.16–39.86)	9.61 (1.14–80.74)	0.75 (0.05–12.11)	6.09 (0.73–51.03)	4.70 (0.48–45.84)

Readmission for severe malaria	30, 50 (44.3)	29, 47 (97.6)	61, 151 (158.5)	75, 129 (87.5)	73, 178 (127.4)	33, 70 (101.7)
		**2.01 (1.34–3.03)**	**2.36 (1.60–3.46)**	**1.84 (1.25–2.72)**	**2.23 (1.53–3.23)**	**1.80 (1.16–2.78)**

Readmission for severe malarial anemia or severe anemia^b,c^	0, 0 (0.0)	4, 6 (12.4)	19, 43 (44.6)	6, 9 (6.1)	27, 53 (37.8)	4, 5 (7.2)
		--	--	--	--	--

Readmission for severe malarial anemia^b,d^	0, 0 (0.0)	4, 6 (12.4)	18, 38 (39.4)	6, 7 (4.7)	23, 46 (32.7)	4, 5 (7.2)
		--	--	--	--	--

Readmission for severe anemia	0, 0 (0.0)	0, 0 (0.0)	3, 5 (5.2)	1, 2 (1.4)	5, 7 (5.0)	0, 0 (0.0)
		--	--	--	--	--

Readmission for other reasons	10, 11 (9.7)	7, 8 (16.5)	13, 19 (19.6)	30, 37 (25.0)	22, 28 (19.9)	13, 19 (27.5)
		1.82 (0.79–4.20)	1.91 (0.89–4.09)	**2.42 (1.29–4.53)**	2.17 (1.11–4.27)	2.45 (1.19–5.05)

Clinic visit for any illness^b^	93, 318 (281.1)	36, 103 (212.8)	73, 193 (198.9)	119, 385 (259.7)	93, 204 (144.8)	54, 177 (255.2)
		1.09 (0.87–1.36)	0.94 (0.78–1.13)	1.05 (0.90–1.23)	0.81 (0.68–0.97)	1.03 (0.85–1.25)

Clinic visit for uncomplicated malaria	37, 62 (54.8)	16, 27 (55.8)	37, 60 (61.8)	54, 84 (56.7)	41, 56 (39.8)	23, 34 (49.0)
		1.65 (0.99–2.72)	1.32 (0.92–1.90)	1.34 (0.94–1.90)	1.19 (0.82–1.73)	1.19 (0.79–1.80)

Clinic visit for illness unrelated to malaria^b^	80, 256 (266.3)	32, 76 (157.0)	61, 133 (137.1)	108, 301 (203.0)	74, 148 (105.1)	48, 143 (206.2)
		1.10 (0.85–1.44)	0.92 (0.74–1.16)	1.08 (0.90–1.30)	0.81 (0.66–0.99)	1.05 (0.85–1.30)

Abbreviations: CC, community children; CM, cerebral malaria; RDS, respiratory distress syndrome; MS, multiple seizures; SMA, severe malarial anemia; Pros, prostration; aHR, adjusted hazard ratio; CI, confidence interval.

a Adjusted hazard ratios (aHR) obtained from PWP-TT analysis for each recurrent outcome comparing severe malaria manifestations to CC, adjusted for age, study site, presence of HIV infection, and socioeconomic status at enrollment. Significant aHR for each outcome following Bonferroni correction for 5 comparisons to CC are bolded (p < 0.01).

After Bonferroni correction for 10 comparisons between severe malaria manifestations for each outcome (p<0.005), the following groups differed:

b MS vs SMA

c SMA vs Pros

d RDS vs MS

## Data Availability

The datasets used and or analyzed in the present study are available upon reasonable request to ccjohn@iu.edu and opokabob@yahoo.com

## Abbreviations

aHR: Adjusted Hazard Ratio
CC: Community Children
CI: Confidence Interval
CM: Cerebral Malaria
HIV: Human Immunodeficiency Virus
MS: Malaria with complicated Seizures
PWP-TT: Prentice-Williams-Peterson Total-Time
RDS: Respiratory Distress Syndrome
RDT: Rapid Diagnostic Test
SES: SocioEconomic Status
SM: Severe Malaria
SMA: Severe Malarial Anemia
USA: United States of America

## References

[1] WHO. World Malaria Report Geneva: WHO; 2019.

[2] Idro R, Aloyo J, Mayende L, Bitarakwate E, John CC, Kivumbi GW. Severe malaria in children in areas with low, moderate and high transmission intensity in Uganda. Tropical medicine & international health : TM & IH. 2006;11(1):115–24. 16398762 10.1111/j.1365-3156.2005.01518.x

[3] Idro R, Marsh K, John CC, Newton CR. Cerebral malaria: mechanisms of brain injury and strategies for improved neurocognitive outcome. Pediatric research. 2010;68(4):267–74. 20606600 10.1203/PDR.0b013e3181eee738 PMC3056312

[4] Obonyo CO, Vulule J, Akhwale WS, Grobbee DE. In-hospital morbidity and mortality due to severe malarial anemia in western Kenya. The American journal of tropical medicine and hygiene. 2007;77(6 Suppl):23–8. 18165471

[5] Biemba G, Dolmans D, Thuma PE, Weiss G, Gordeuk VR. Severe anaemia in Zambian children with Plasmodium falciparum malaria. Trop Med Int Health. 2000;5(1):9–16. 10672200 10.1046/j.1365-3156.2000.00506.x

[6] Taylor T, Olola C, Valim C, Agbenyega T, Kremsner P, Krishna S, Standardized data collection for multi-center clinical studies of severe malaria in African children: establishing the SMAC network. Trans R Soc Trop Med Hyg. 2006;100(7):615–22. 16551469 10.1016/j.trstmh.2005.09.021 PMC1459261

[7] Phiri KS, Calis JC, Faragher B, Nkhoma E, Ng’oma K, Mangochi B, Long term outcome of severe anaemia in Malawian children. PLoS One. 2008;3(8):e2903. 18682797 10.1371/journal.pone.0002903 PMC2488370

[8] Zucker JR, Lackritz EM, Ruebush TK, 2nd, Hightower AW, Adungosi JE, Were JB, Childhood mortality during and after hospitalization in western Kenya: effect of malaria treatment regimens. Am J Trop Med Hyg. 1996;55(6):655–60. 9025694 10.4269/ajtmh.1996.55.655

[9] Lackritz EM, Hightower AW, Zucker JR, Ruebush TK, 2nd, Onudi CO, Steketee RW, Longitudinal evaluation of severely anemic children in Kenya: the effect of transfusion on mortality and hematologic recovery. Aids. 1997;11(12):1487–94. 9342071 10.1097/00002030-199712000-00013

[10] Opoka RO, Hamre KES, Brand N, Bangirana P, Idro R, John CC. High Postdischarge Morbidity in Ugandan Children With Severe Malarial Anemia or Cerebral Malaria. J Pediatric Infect Dis Soc. 2017;6(3):e41–e8. 28339598 10.1093/jpids/piw060 PMC5907851

[11] UBOS. Uganda Demographic and Health Survey, 2016. Kampala, Uganda 2016.

[12] Bangirana P, John CC, Idro R, Opoka RO, Byarugaba J, Jurek AM, Socioeconomic predictors of cognition in Ugandan children: implications for community interventions. PLoS One. 2009;4(11):e7898–e. 19936066 10.1371/journal.pone.0007898 PMC2774512

[13] Westbury LD, Syddall HE, Simmonds SJ, Cooper C, Sayer AA. Identification of risk factors for hospital admission using multiple-failure survival models: a toolkit for researchers. BMC Med Res Methodol. 2016;16:46. 27117081 10.1186/s12874-016-0147-x PMC4845493

[14] Opoka RO, Waiswa A, Harriet N, John CC, Tumwine JK, Karamagi C. Blackwater Fever in Ugandan Children With Severe Anemia is Associated With Poor Postdischarge Outcomes: A Prospective Cohort Study. Clinical infectious diseases : an official publication of the Infectious Diseases Society of America. 2020;70(11):2247–54. 10.1093/cid/ciz648 PMC7245149 31300826

[15] Kwambai TK, Dhabangi A, Idro R, Opoka R, Watson V, Kariuki S, Malaria Chemoprevention in the Postdischarge Management of Severe Anemia. N Engl J Med. 2020;383(23):2242–54. 33264546 10.1056/NEJMoa2002820 PMC9115866

[16] Newton CR, Krishna S. Severe falciparum malaria in children: current understanding of pathophysiology and supportive treatment. Pharmacol Ther. 1998;79(1):1–53. 9719344 10.1016/s0163-7258(98)00008-4

[17] van Hensbroek MB, Palmer A, Jaffar S, Schneider G, Kwiatkowski D. Residual neurologic sequelae after childhood cerebral malaria. J Pediatr. 1997;131(1 Pt 1):125–9. 9255203 10.1016/s0022-3476(97)70135-5

[18] John CC, Bangirana P, Byarugaba J, Opoka RO, Idro R, Jurek AM, Cerebral malaria in children is associated with long-term cognitive impairment. Pediatrics. 2008;122(1):e92–9. 18541616 10.1542/peds.2007-3709 PMC2607241

[19] Carter JA, Ross AJ, Neville BG, Obiero E, Katana K, Mung’ala-Odera V, Developmental impairments following severe falciparum malaria in children. Trop Med Int Health. 2005;10(1):3–10. 15655008 10.1111/j.1365-3156.2004.01345.x

[20] Ngoungou EB, Preux PM. Cerebral malaria and epilepsy. Epilepsia. 2008;49 Suppl 6:19–24. 10.1111/j.1528-1167.2008.01752.x 18754957

[21] Idro R, Kakooza-Mwesige A, Asea B, Ssebyala K, Bangirana P, Opoka RO, Cerebral malaria is associated with long-term mental health disorders: a cross sectional survey of a long-term cohort. Malaria journal. 2016;15:184. 27030124 10.1186/s12936-016-1233-6 PMC4815157

[22] Idro R, Kakooza-Mwesige A, Balyejjussa S, Mirembe G, Mugasha C, Tugumisirize J, Severe neurological sequelae and behaviour problems after cerebral malaria in Ugandan children. BMC research notes. 2010;3:104. 20398391 10.1186/1756-0500-3-104 PMC2861066

[23] Ssenkusu JM, Hodges JS, Opoka RO, Idro R, Shapiro E, John CC, Long-term Behavioral Problems in Children With Severe Malaria. Pediatrics. 2016;138(5). 10.1542/peds.2016-1965 PMC5079082 27940786

[24] Bangirana P, Opoka RO, Boivin MJ, Idro R, Hodges JS, Romero RA, Severe malarial anemia is associated with long-term neurocognitive impairment. Clin Infect Dis. 2014;59(3):336–44. 24771329 10.1093/cid/ciu293 PMC4155441

[25] Byakika-Kibwika P, Nyakato P, Lamorde M, Kiragga AN. Assessment of parasite clearance following treatment of severe malaria with intravenous artesunate in Ugandan children enrolled in a randomized controlled clinical trial. Malar J. 2018;17(1):400. 30376860 10.1186/s12936-018-2552-6 PMC6208070

[26] Byakika-Kibwika P, Achan J, Lamorde M, Karera-Gonahasa C, Kiragga AN, Mayanja-Kizza H, Intravenous artesunate plus Artemisnin based Combination Therapy (ACT) or intravenous quinine plus ACT for treatment of severe malaria in Ugandan children: a randomized controlled clinical trial. BMC Infect Dis. 2017;17(1):794. 29281988 10.1186/s12879-017-2924-5 PMC5745850

[27] Bukirwa H, Yeka A, Kamya MR, Talisuna A, Banek K, Bakyaita N, Artemisinin combination therapies for treatment of uncomplicated malaria in Uganda. PLoS clinical trials. 2006;1(1):e7. 16871329 10.1371/journal.pctr.0010007 PMC1488893

[28] Phiri K, Esan M, van Hensbroek MB, Khairallah C, Faragher B, ter Kuile FO. Intermittent preventive therapy for malaria with monthly artemether-lumefantrine for the post-discharge management of severe anaemia in children aged 4–59 months in southern Malawi: a multicentre, randomised, placebo-controlled trial. Lancet Infect Dis. 2012;12(3):191–200. 22172305 10.1016/S1473-3099(11)70320-6

[29] Organization WH. WHO guidelines for malaria, 3 June 2022. World Health Organization; 2022.

